# DeepDynamicHand: A Deep Neural Architecture for Labeling Hand Manipulation Strategies in Video Sources Exploiting Temporal Information

**DOI:** 10.3389/fnbot.2018.00086

**Published:** 2018-12-17

**Authors:** Visar Arapi, Cosimo Della Santina, Davide Bacciu, Matteo Bianchi, Antonio Bicchi

**Affiliations:** ^1^Centro di Ricerca “Enrico Piaggio,” Università di Pisa, Pisa, Italy; ^2^Dipartimento di Ingegneria dell'Informazione, Università di Pisa, Pisa, Italy; ^3^Dipartimento di Informatica, Università di Pisa, Pisa, Italy; ^4^Soft Robotics for Human Cooperation and Rehabilitation, Fondazione Istituto Italiano di Tecnologia, Genova, Italy

**Keywords:** hand detection, gesture recognition, manipulation action, human grasping, deep leaning, convolutional and recurrent neural networks, robotics

## Abstract

Humans are capable of complex manipulation interactions with the environment, relying on the intrinsic adaptability and compliance of their hands. Recently, soft robotic manipulation has attempted to reproduce such an extraordinary behavior, through the design of deformable yet robust end-effectors. To this goal, the investigation of human behavior has become crucial to correctly inform technological developments of robotic hands that can successfully exploit environmental constraint as humans actually do. Among the different tools robotics can leverage on to achieve this objective, deep learning has emerged as a promising approach for the study and then the implementation of neuro-scientific observations on the artificial side. However, current approaches tend to neglect the dynamic nature of hand pose recognition problems, limiting the effectiveness of these techniques in identifying sequences of manipulation primitives underpinning action generation, e.g., during purposeful interaction with the environment. In this work, we propose a vision-based supervised Hand Pose Recognition method which, for the first time, takes into account temporal information to identify meaningful sequences of actions in grasping and manipulation tasks. More specifically, we apply Deep Neural Networks to automatically learn features from hand posture images that consist of frames extracted from grasping and manipulation task videos with objects and external environmental constraints. For training purposes, videos are divided into intervals, each associated to a specific action by a human supervisor. The proposed algorithm combines a Convolutional Neural Network to detect the hand within each video frame and a Recurrent Neural Network to predict the hand action in the current frame, while taking into consideration the history of actions performed in the previous frames. Experimental validation has been performed on two datasets of dynamic hand-centric strategies, where subjects regularly interact with objects and environment. Proposed architecture achieved a very good classification accuracy on both datasets, reaching performance up to 94%, and outperforming state of the art techniques. The outcomes of this study can be successfully applied to robotics, e.g., for planning and control of soft anthropomorphic manipulators.

## 1. Introduction

The hand is the primary tool humans rely on to successfully interact with the external environment, capitalizing on the intrinsic adaptability of human “end-effector” to multiply manipulation and grasping capabilities. On the robotic side, human inspiration could be the successful strategy for the design and control of robotic manipulators, which can deform to purposefully exploit the environmental constraints (Deimel and Brock, 2016) as humans actually do. For the reasons above, a correct recognition of hand pose and gesture represents an active field of research with applications that are not limited to human-robot interaction and human-inspired robotic grasping (Terlemez et al., 2014) but cross-fertilize several technological and scientific domains, such as neuroscience (Santello et al., 2016), rehabilitation (Dipietro et al., 2008), tele-operation (Fani et al., 2018), haptics and virtual reality (Bianchi et al., 2013), just to cite a few. The robotics interest in filling the gap between human performance and artificial results has also motivated the creation of manipulation and object datasets, with the goal of favoring benchmarking, devising grasp planning as well as robotic design and control strategies. For a review on this topic please refer to Huang et al. (2016).

In literature, hand pose recognition is usually performed through wearable or remote devices (Ciotti et al., 2016). The former category comprises glove and surface marker-based systems. We refer the interested reader to Dipietro et al. (2008) for a comprehensive survey about this topic. Regarding remote systems, video recording represents the most commonly used strategy. Indeed, there exists vast literature where video and/or photo information is processed for gesture recognition, relying on the usage of different techniques, such as of k-means (Ong and Bowden, 2004), penalized maximum likelihood estimation (Cheng et al., 2012), and hidden Markov models (Beh et al., 2014). For a comparative analysis of these techniques please refer to Rautaray and Agrawal (2015).

Deep learning have recently attracted an ever-expanding interest in sever application fields dealing with the necessity of making sense of complex, raw and noisy data. Convolutional Neural Networks (CNN) (LeCun et al., 1998), for instance, have demonstrated to be an effective class of neural models for image recognition (Simonyan and Zisserman, 2014), segmentation (LeCun et al., 2015), feature detection (Girshick et al., 2014) and retrieval applications (Babenko et al., 2014; Zeiler and Fergus, 2014), by scaling up the networks to tens of millions of parameters, and capitalizing upon massive labeled datasets to support the learning process (e.g., Huang et al., 2016). When focusing on CNN applications to hand gesture recognition, pioneer works date back to 90s (Nowlan and Platt, 1995), while more recent approaches use weakly labeled data to train the network for continuous sign language recognition (Koller et al., 2016). In Bambach et al. (2015) authors presented EgoHands, a CNN trained on 4,800 egocentric images (i.e., captured through a wearable first-person camera) of multiple people interacting with each other. EgoHands is able to accurately detect and segment one or more hands from static images, with very robust performances with respect to changes in light and environment conditions, especially during Environmental Constraint Exploitation (ECE) (Eppner et al., 2015) for grasping.

The main limitation shared by works in literature that deal with hand pose and action estimation is that they usually consider only static information. This limitation is especially critical when considering robotic applications, such as data-driven human-inspired robotic planning, where the dynamic content of the action is crucial for a correct and successful task execution. To the authors' best knowledge no work has focused yet on automatic classification of sequences of human hand action primitives from video, that can account for the dynamic time-dependent information. This occurs despite the fact that human hand behavior is intrinsically time dependent (Haken et al., 1985). In this respect, the EgoHands model (Bambach et al., 2015) handles a restricted form of dynamic information, by allowing to recognize a single activity per video using dynamic frame information, while the classification of different actions combined in a single visual stream is not permitted.

In this paper we propose a neural approach, named DeepDynamicHand, with the goal of extracting human hand dynamic information during the interaction with the environment. Main motivation for this work is the increasing attention—as previoulsy mentioned—for the design of soft robotic hands (Della Santina et al., 2018), which embody the ability to comply and adapt to environmental characteristics for manipulation, in a human-inspired fashion. DeepDynamicHand comprises two neural components. The first is a CNN model, pre-trained to segment human hands in images (Bambach et al., 2015), which extracts a compressed and rich representation of the hand posture information for each video frame. The second component is a Recurrent Neural Network (RNN), implemented using Long Short Term Memory (LSTM) recurrent units (Hochreiter and Schmidhuber, 1997), which receives in input the sequence of CNN encodings for the videos. The LSTM is then trained to predict the sequences of action primitives—i.e., a dictionary of meaningful behavior components whose composition enables to successfully interact with the environment in grasping tasks—performed by human hands in the video.

We have tested the effectiveness of the proposed approach on two datasets. The first one comprises third-person non egocentric videos fully frame-by-frame human labeled to identify ECE hand action primitives (Eppner et al., 2015; Puhlmann et al., 2016). DeepDynamicHand was proven to be able to classify the dynamic hand strategies of this dataset with an accuracy up to 94%. These results appear very encouraging given the poor definition and coloring of the frames under investigation and the highly dynamical and complex manipulation strategies involving the interaction with the environment. The second benchmark serves to assess the effectiveness of our approach comparatively with state of the art methods and it is based on the dataset used to train the EgoHands CNN in Bambach et al. (2015). The results of our analysis reveal that DeepDynamicHand outperforms the EgoHand in the task of classifying daily-life activities, such as playing cards, even when restricting to a simplified scenario comprising a single activity for each video stream. The impact of the translation of these results on the robotic side, especially for the design of soft robotic hands that exploit EC as humans do are finally discussed.

## 2. Related Work

In the past few years, deep learning has demonstrated to be an effective tool for image classification (Simonyan and Zisserman, 2014), object detection (Girshick et al., 2014), as well as face and hand recognition tasks (Bambach et al., 2015; Parkhi et al., 2015). CNNs are currently the most popular building block for machine vision applications, thanks to their ability of effectively processing raw data, automating the feature extraction process that before required heavily hand-engineered procedures. When it comes to CNN applications in robotic manipulation, (Yang et al., 2015) proposed a system to learn manipulation actions by processing unconstrained videos. The system consists of two CNN-based recognition modules, one for classifying the hand grasp type and the other for object recognition. The manipulation action is hence learned and generated through a grammar parser module, which combines both the objects and the left and right hand grasping types. In this work, dynamic information on temporal correlation between video frames is not considered. In Bambach et al. (2015), it has been developed a hand-based activity recognition method to classify whole labeled video frames, to identify one among four activity types (playing cards, chess, Jenga and solving puzzle). To incorporate temporal dependencies, each frame is classified using a fixed-size temporal window centered on the frame. Results show that temporal information significantly increases the accuracy of activity recognition, even though a simple voting-based approach is used. Furthermore, the classification is intended on the whole video sequence, while the dynamic action components underpinning the complete task execution cannot be identified.

To enable a successful exploitation and learning of robust spatio-temporal features, CNNs are often combined with RNN (Karpathy et al., 2014; Ng et al., 2015; Nguyen et al., 2017), typically exploiting LSTM recurrent cells which allow to store a compressed representation of medium-range temporal relationships in the input data.

For instance, in the context of video captioning applications, Nguyen et al. (2017) proposed a method that combines a CNN to extract visual features from the video frames and two LSTM layers to generate a network prediction defined as a list of words describing hand grasping and object types. The visual features are frame-oriented: this means that there is no explicit information as concerns hand poses, neither any temporal sequence to characterize the dynamics of the hand movement and pose evolution. Such static descriptions are interesting for the analysis of contact configurations between the hand and objects but the dynamics of the action is not taken into account by this model.

In Wang et al. (2018) the authors used a combination of autoencoders and support vector machine classifiers to automatically recognize everyday human activities (such as driving, walking, etc). However, the dynamic segmentation of action primitives, as we target in this paper for grasping, was out of the scope of the work. In Garcia-Hernando et al. (2017) a model for hand-pose estimation was proposed, which employed, in addition to RGB data (as we do in our work), depth information. In Sudhakaran and Lanz (2017) a combined usage of CNN and RNN, similar to the one we present in this manuscript, was proposed for the recognition of first person perspective interactions. The dataset they used to validate their architecture consists of first person interaction videos: each video contains one activity execution among different types of activities. Very recently, in Zhang Y. et al. (2018), a new benchmark dataset was released to evaluate state-of-the-art deep neural networks for activity recognition from egocentric videos. The focus was to recognize daily actions rather than manipulation primitives and the presence of depth information together with luminance and color was assumed. This consistently restricts the range of processable videos with respect to our general approach (intended to analyze videos from non-specialist sources such as YouTube).

To the authors' best knowledge, our work is the first that applies machine learning techniques to extract dynamic time-related information of human hand poses from videos involving complex interactions of the hand with the environment. More specifically, the visual features are hand centric and the videos we use are labeled in a dynamic fashion, taking into account both pre-grasp and grasping actions.

## 3. Problem Definition

We tackle the problem of converting human object manipulation and grasping videos into a sequence of *action primitives*. With the term action primitive we refer to a set of patterns describing specific meaningful task-oriented behavior of human hand in manipulation tasks. Let us consider a collection of videos

(1) $$<V_{1},V_{2},\cdots ,V_{T}>$$

Each video **V**_*j*_ is composed by a sequence of frames

(2) $$V_{j}=(I_{j,1},I_{j,2},\cdots ,I_{j,n})$$

where $I_{j,t}\in {\mathbb{R}}^{w\times h\times 3}$, *w* and *h* are the number of pixels (width and height respectively) for the image captured at frame *t*. Note that the video length *n* will, in general, vary from sample to sample. Our objective is to generate a sequence of labels corresponding to action primitives

(3) $$Y_{j}=(y_{j,1},y_{j,2},\cdots ,y_{j,n})$$

where given label $y_{j,t}\in {\mathbb{R}}^{\left|D\right|}$, the subscripts *j* and *t* refer to video and temporal frame, respectively. The set *D* is a pre-defined (finite) human label dictionary, whose level of granularity depends on the task under investigation. Our aim, hence, is to automatically generate a frame by frame labeling, where the label represents the information regarding the hand pose we want to infer from each frame. Furthermore, since a video is characterized by a dynamic temporal structure, the labels composing the sequence must comply with temporal constraints. In other words, we are in a scenario where the action **y**_*j,t*_ associated to a frame *t* depends on the information present both in the current as well as in previous frames (**I**_*j,t*_, **I**_*j,t*−1_, …, **I**_*j*,1_).

The problem we treat stems from the observation that complex interactions between hand, environment and objects usually consist of a sequence of steps or events. Discovering these underlying steps as latent events is mandatory not only to give insights on human motor control strategies but also to favor a direct translation of neuroscientific observations to robotics. Each event is characterized by two main components: (i) the target the hand achieves at the end of the event, and (ii) the pose patterns the hand follows during this event. Since labels, hence action primitives, are task oriented, the action primitive may represent both the event (defining the atomic behavior of the hand during a grasp strategy such as in Puhlmann et al. (2016; e.g., approaching, closing, flipping etc.) or a set of events (incorporating them in order to recognize an entire activity associated to a complete video, e.g., game board activity such as playing chess, playing Jenga etc.).

## 4. Our Approach

**Figure 1:**
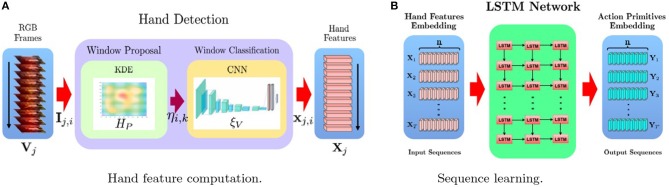
An overview of our proposed framework. IIn the first stage **(A)**, a CNN based technique is used to detect hands in video frames, encoding the corresponding bounding boxes into feature vectors corresponding to the activations of the last fully connected layer of the CNN. In the second stage **(B)**, an LSTM model is used to learn to classify the sequence of *n* input vectors, corresponding to the *n* video frames, into a sequence of *n* hand action primitives.

In this paper we propose a two-stage framework (Figure 1). Let us consider a generic video **V**_*j*_, the aim of the first stage (Figure 1A) is to detect the hand in each video frame **I**_*j,i*_ and to extract a summarized and, yet, informative characterization of the hand pose. For this purpose, we leverage on two modules. The *window proposal* module

(4) $$H_{P}:{\mathbb{R}}^{w\times h\times 3}\to {\mathbb{R}}^{w_{k}\times h_{k}\times 3},$$

where *w*_*k*_ ≤ *w* and *h*_*k*_ ≤ *h*, generates candidate bounding boxes that are likely to contain the hand. These are passed to the *window classification* module

(5) $$\xi_{V}:{\mathbb{R}}^{w_{k}\times h_{k}\times 3}\to (p,1-p)\in {\mathbb{R}}^{2},$$

where *p* ∈ [0, 1], whose aim is to determine if a hand is present in the candidate box.

Letting $\eta_{i,k}\in {\mathbb{R}}^{w_{k}\times h_{k}\times 3}$ be the candidate bounding box which is scored as the most likely to contain a hand, we then use the model ξ_*V*_ to obtain a fixed-length vectorial encoding of the hand pose features $x_{j,i}\in {\mathbb{R}}^{g}$. The whole video **V**_*j*_ can now be represented as sequence **X**_*j*_ = (**x**_*j*,1_, **x**_*j*,2_, …, **x**_*j,n*_) of such hand-pose encodings, one for each video frame. In the second stage (Figure 1B), the *the sequence learning* model is trained to process **X**_*j*_ to generate a corresponding output sequence **Y**_*j*_, containing the action primitive performed by the hand in each frame. The key point is that the sequence model is able to output the prediction for the current frame while taking into account the history of the poses and action sequences performed by the hand in the previous instants.

## 5. Hand Feature Computation

In this section we describe in detail the single modules that represent the pillars of the *hand feature computation* part. We propose to process videos off-line to learn patterns describing specific meaningful task-oriented behavior of human hand in manipulation tasks.

### 5.1. Hand Detection

Hand detection in a single video frame **I**_*j,i*_ is a two-step process: first, we generate several candidate bounding boxes; then, such boxes are fed to a pre-trained CNN that classifies them based on weather they contain or not a hand. The input of this whole block is the video frame **I**_*j,i*_, while the output is the vector containing the hand-presence probability for each of the candidate bounding boxes.

#### 5.1.1. Window Proposal

Proposal methods allow to focus processing only on the most salient and distinctive locations of an image, reducing the computational cost of the detection task by limiting the area on which this is performed. Window proposal techniques (Hosang et al., 2016) have emerged mainly in object identification, where candidate bounding boxes are scored according to how likely they are to contain an object. When focusing on hand detection, there are hand location and size biases which can be considered (Lee et al., 2014). In our work, we adopt the method proposed in Bambach et al. (2015) to obtain a sample of candidate windows that combines spatial biases and appearance models in a unified probabilistic framework. The goal is to model the probability that the hand appears in a certain region of the image, using such a distribution to propose a small set of most likely regions where the hand can occur. For this purpose we use the pre-trained four-dimensional Gaussian Kernel Density estimator (KDE) fitted on the *EgoHands* dataset (Bambach et al., 2015). A measure to evaluate window proposal methods is given by its *coverage* − i.e., the percentage of ground truth objects that have a high enough overlap (intersection over union) with the proposed windows to be counted as positive during detection.

#### 5.1.2. Window Classification

We can now use a CNN to classify each candidate window obtained in the previous step, by associating it with the probability of containing a hand. Initial CNNs have been inspired by the structure of the mammals visual processing system but have recently evolved into complex multi-layered computational models whose constituents have often little biological plausibility.

The key operator of CNN is convolution which is a means by which the network parameters can be contained, by having the image pixels share parameters with each other, while introducing some translation invariance. Discrete two dimensional convolution between an image *I* and a kernel *K* is defined by

$$\left(I*K)(i,j)=\sum_{m\;=\;1}^{M}\sum_{n\;=\;1}^{N}I(i-m,j-m)K(m,n\right)$$

where *K* is basically an *M* × *N* matrix containing the convolutional parameters (learned by the training algorithm).

The structure of a “classical” CNN (Krizhevsky et al., 2012) is made by a number of *convolutional layers* (Conv), each comprising a bank of *K* kernels whose size and number can vary between layers. Since convolution is a linear operator, its output is typically passed through some form of (weak) non-linearity, the most common being the Rectified Linear Unit (ReLU) *f*(*x*) = *max*(0, *x*), a computationally efficient approximation to saturating sigmoid nonlinearities with excellent properties in terms of robustness to gradient vanishing. The result of the non-linearly filtered convolution is either fed to a new convolutional layer or it is given in input to a *pooling layer* (Pool). This layer combines the values in a neighborhood of pixels defined by a mask (typically a 3 × 3) through a pooling operation (typically a max function) intended to enforce invariance and to subsample the image to allow performing multi-resolution analysis at further stages. The use of unbounded ReLu activation and max pooling yields to potentially unbounded activations: hence, the use of *normalization layers* (Norm) is also common. The sparse convolutional layers are completed by a variable number of *fully connected* (FC) layers, aggregating into a single vector all local features (relative to input image such as edges, blobs, shapes, etc.) held separately in the previous layers.

**Figure 2:**
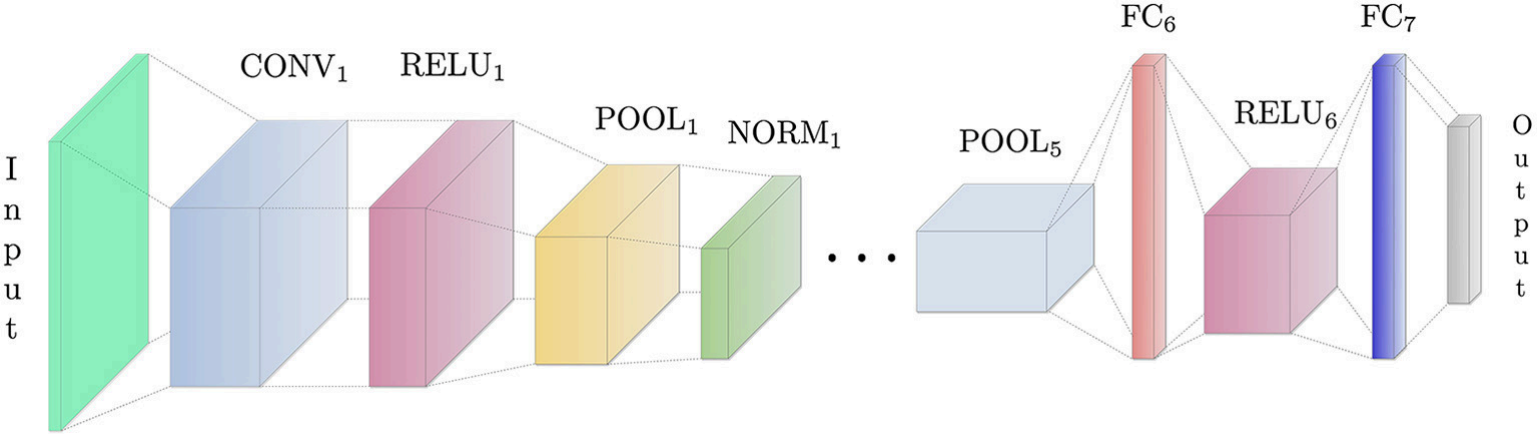
Condensed view of the CNN architecture used in this work (note that, for the sake of clarity, we do not show all the layers of the network). The CNN has a standard structure comprising a combination of convolutional layers (applying a sliding dot-product between the convolution kernels and the image), ReLU layers (introducing non-linearity after the linear convolution operations), pooling layers (downsampling convolutional values by taking the maxim with respect to a small neighborhood of each pixel), normalization layers (normalizing unbounded activations) and fully connected layers (connecting every neuron in one layer to every neuron in another layer). The final layer is connected to a softmax classifier.

**Figure 3:**
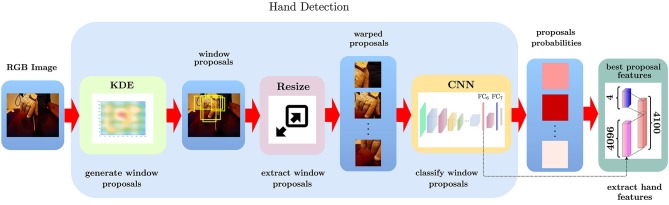
Detailed procedure to compute the hand pose encoding for a frame. Given an input RGB video frame, we first generate candidate windows that are likely to contain the hand; then, we resize and subtract the global mean for each candidate window to make it suitable for CNN processing. Then a CNN is used to classify each candidate window as containing or not a hand. Finally, we extract the features from the *FC6* CNN's layer and combine them with the coordinate and width and height relative to the best candidate to generate the final frame encoding.

In this work, we use the pre-trained CNN (Figure 2) developed as part of the EgoHands project (Bambach et al., 2015), using it to accurately and robustly detect hands in static video frames. After generating window proposals for each video frame, we resize them since the network takes a single image of fixed size ℝ^*l*×*l*×3^ (*l* = 227 represents both width and height) as input. This image is then processed by five Conv layers, each of them followed both by combinations of ReLU, Pool and Norm layers or only ReLU layers. At the top of the architecture there are two fully connected layers, which take as input the output of the previous layer and applies to it a matrix-vector product. The penultimate fully connected layer also applies a rectified linear transform. The full CNN architecture is illustrated in Table 1. Conv_i_ indicates a convolutional layer with *b* filters of spatial size *f* × *f*, applied to the input with stride *s*. The pooling layer Pool_i_ spatially pools in non-overlapping *f* × *f* input regions with stride *s*; the ReLU_i_ layer introduces non-linearity into the CNN and the normalization layer Norm_i_ normalizes the unbounded activations introduced by previous layer. The FC_i_(n) layer connects all neurons of previous layer with *n* nodes. The output layer of the CNN is a *Softmax classifier* that predicts the probability distribution over two classes (*hand, no-hand*) given the input image. In summary, given a single RGB video frame **I**_*j,i*_, we can itemize the full hand detection pipeline (Figure 3) as follows:

1. generate spatially sampled window proposals $\eta_{i,k}\in {\mathbb{R}}^{w_{k}\times h_{k}\times 3}$, and represent each of them by image coordinate, i.e., width and height in terms of the bounding boxes;
2. resize each window to 227 × 227 × 3 pixels and normalize by subtracting the mean of the Egohands training data (Bambach et al., 2015);
3. classify the window crops with the pre-trained CNN using *CaffeNet* (Jia et al., 2014).

**Table 1:** Description of the structure of the CNN.

**Type**	**Kernels (*b*)**	**Spatial size (*f*)**	**Stride (*s*)**	**Output size**
Conv_1_	96	11	4	55 × 55 × 96
ReLU_1_	–	–	–	55 × 55 × 96
Pool_1_	–	3	2	27 × 27 × 96
Norm_1_	–	–	–	27 × 27 × 96
Conv_2_	256	5	1	27 × 27 × 256
ReLU_2_	–	–	–	27 × 27 × 256
Pool_2_	–	3	2	13 × 13 × 256
Norm_2_	–	–	–	13 × 13 × 256
Conv_3_	384	3	1	13 × 13 × 384
Conv_4_	384	3	1	13 × 13 × 384
ReLU_4_	–	–	–	13 × 13 × 384
Conv_5_	256	3	1	13 × 13 × 256
ReLU_5_	–	–	–	13 × 13 × 256
Pool_5_	–	3	2	6 × 6 × 256
FC_6_	–	–	–	4096
ReLU_6_	–	–	–	4096
FC_7_	–	–	–	4096

*Each row describes a layer of the network, organized from input to output. Kernels refers to the number (b) of kernels (each of them is a matrix f × f whose dimensions are determined by the spatial size f) containing the convolutional parameters, stride controls the shift step of the kernel (convolution layer) or the mask (pooling layer) around the input volume, and output size represents the output dimension (height, weight and depth in the case of a volume or the vector size)*.

The time computation required by the CNN to classify each window proposal is 10 ms on a NVIDIA Tesla M40 GPU with 12 GB of onboard memory. The time required to detect the hand instead depends on the number of window proposals generated for each video frame. Such number should be enough to ensure a reasonable tradeoff between the computation time and the coverage of the entire image. In our experiments we generated 500 window proposals per image, consequently the total time requested to detect the hand in each video frame is 5 s.

### 5.2. Hand Feature Extraction

It is important to underline that convolution, pooling, normalization and ReLu layers are the basic building blocks of the CNN as shown in Figure 2. These layers allow to extract the useful features associated to the presence of the hand in the image, introducing non-linearity in the network and reducing feature dimension, while making feature extraction robust to scaling and translation. The output volume of the last pooling layer Pool_5_ acts as an input to the first fully connected layer FC_6_. The term “fully connected” implies that every neuron in the previous layer is connected to every neuron on the next layer. Since the output from the convolutional and pooling layers represents high-level features of the input image, the purpose of the fully connected layer is to summarize them into a fixed-length vectorial encoding that can be effectively used as image signature to ultimately perform the prediction (e.g., a classification task for object recognition in the image). The intuition is that by adding a fully connected layer one allows an adaptive non-linear combination of the features discovered by the convolutional layer. To retrieve hand features we do not use the CNN output as it is, but we extract information from its intermediate layers since the activation of the fully connected layers is enough to characterize the hand pose. The EgoHands CNN we use in this work contains two FC layers. We consider layer FC_6_ as it is characterized by a less task-specific encoding than FC_7_, which is closer to the network output and hence more specialized on the hand-detection task. Ultimately, the hand pose representation for the frame extracted by layer FC_6_ is a vector $x_{FC_{6}}\in {\mathbb{R}}^{n}$ (where *n* = 4,096). In the last block of Figure 3, we show the final hand pose features, i.e., a vector $x_{i}\in {\mathbb{R}}^{g}$ (where *g* = 4,100), where the first four components are bounding box coordinates, width and height, while the remainder contain the **x**_*F*_*C*__6__ vector.

## 6. Sequence Learning

In this section, we describe in detail the *sequence learning* part (introduced here for the first time), the underlying LSTM network (Hochreiter and Schmidhuber, 1997) and its use with the hand features described in the previous section and the action primitives we would like to predict from the videos.

**Figure 4:**
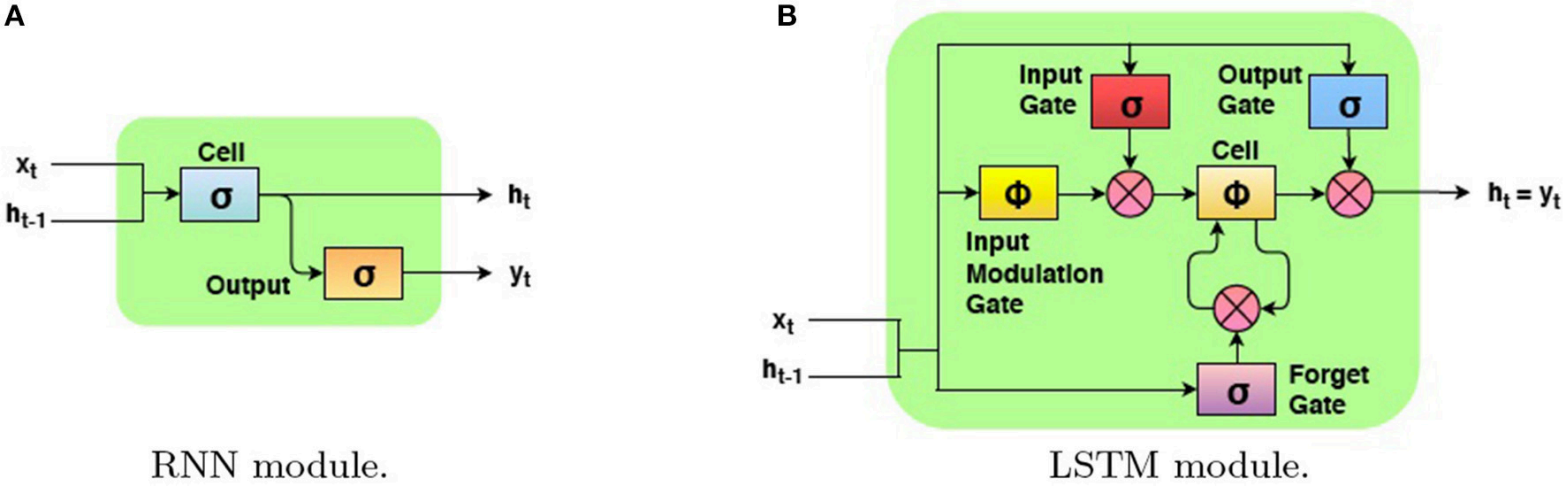
A diagram of a basic RNN module **(A)** and an LSTM module **(B)**.

RNNs are a class of neural networks that maintains an internal representation of the temporal behavior of sequences with arbitrary lengths through directed cyclic connections between its units. Differently from an hidden Markov model, it uses nonlinear transition functions to model long term temporal dependencies by mapping input sequences to a sequence of hidden states, and hidden states to outputs (Figure 4A). More formally, given an input sequence of features **X** = (**x**_1_, **x**_2_, …, **x**_*n*_) (where **x**_*i*_ represents a hand feature vector at frame tth), a RNN computes the corresponding hidden vector sequence **h** = (**h**_1_, **h**_2_, …, **h**_*n*_) and output vector sequence **Y** = (**y**_1_, **y**_2_, …, **y**_*n*_) (where **y**_*i*_ represents label at frame tth) by iterating the following equations from *t* = 1 to *n*:

(6) $$\begin{array}{l} h_{t}=\sigma (W_{xh}x_{t}+W_{hh}h_{t-1}+b_{h})\\ y_{t}=\sigma (W_{hy}h_{t}+b_{y}). \end{array}$$

In (6) σ:ℝ ↦ [0, 1] is an element-wise non-linearity (a sigmoid), the **W** terms denote weight matrices (e.g., **W**_*xh*_ is the input-hidden weight matrix), the **b** terms refer to bias vectors (e.g., **b**_*h*_ is the hidden bias vector), $x_{t}\in {\mathbb{R}}^{g}$ is the input, $h_{t}\in {\mathbb{R}}^{N}$ is the hidden state with *N* hidden units, and $y_{t}\in {\mathbb{R}}^{\left|D\right|}$ is the output corresponding to frame *t*.

For the purpose of this work, we use a specific type of recurrent units, the LSTM cells (Hochreiter and Schmidhuber, 1997), which are characterized by the presence of articulated gating mechanisms introduced to capture longer temporal dependencies and, overall, to control the gradient vanish/esplosion problem (Pascanu et al., 2013). The core element of an LSTM network is the memory cell *c*, an empowered version of the recurrent neuron which includes an number of gate mechanism to read and write access to the memory, as well as its fading over time. Following the notation in Zaremba and Sutskever (2014) as shown in (Figure 4B), the LSTM unit receives an input **x**_*t*_ at each time step *t*, and computes the hidden state **h**_*t*_ and the memory cell state **c**_*t*_ as follows:

(7) $$\begin{array}{l} i_{t}=\sigma (W_{xi}x_{t}+W_{hi}h_{t-1}+b_{i})\\ f_{t}=\sigma (W_{xf}x_{t}+W_{hf}h_{t-1}+b_{f})\\ o_{t}=\sigma (W_{xo}x_{t}+W_{ho}h_{t-1}+b_{o})\\ g_{t}=\phi (W_{xg}x_{t}+W_{hg}h_{t-1}+b_{g})\\ c_{t}=f_{t}⊙c_{t-1}+i_{t}⊙g_{t}\\ h_{t}=o_{t}⊙\phi (c_{t})\\ \sigma (x)=\frac{1}{1+e^{-x}}\\ \phi (x)=\frac{e^{x}-e^{-x}}{e^{x}+e^{-x}}. \end{array}$$

The symbol ⊙ represents element-wise multiplication while σ:ℝ ↦ [0, 1] and ϕ:ℝ ↦ [−1, 1] are sigmoid and the hyperbolic tangent non-linearities, respectively. The weight **W** and bias **b** are trainable model parameters. In addition to the hidden activation **h**_*t*_ of a standard RNN, the LSTM includes an input gate **i**_*t*_, a forget gate **f**_*t*_ and an output gate **o**_*t*_, plus some auxiliary information such as the input modulation gate **g**_*t*_, and the memory cell value **c**_*t*_.

The sequence learning component of our architecture is realized by a recurrent network comprising LSTM units that, in input, receives the sequence of hand pose encodings **x**_*i*_ obtained by the CNN for each video frame. The LSTM is trained to predict, as an output, the action primitive corresponding to the current frame. To this end, the action primitives (which are symbol from a discrete and finite alphabet) are transformed to a numeric vector using a *one-hot* encoding. Such an encoding represents the *k*th symbol of the action primitive alphabet as a vector of length equal to the alphabet size, where only the *k*th element is set to 1 while the rest is equal to zero. More formally, the one-hot encoding of the action label for frame **x**_*i*_ is vector $y_{i}\in {\mathbb{R}}^{\left|D\right|}$ defined as

(8) $$y_{i}{}^{k}=\{\begin{array}{ll} 1, & \text{if }k=\text{ind}(y_{i})\\ 0, & \text{otherwise}. \end{array}$$

where ind(**y_i_**) is the index of the current label in the dictionary *D*.

The final details of the LSTM network are determined by model selection, using validation data outside of the training and test samples for ensuring robustness and avoiding results biased toward high precision on the test-set. Such final details include, for instance, the number of hidden layers, the number of LSTM units in each layer. The following experimental analysis provides details on the final configuration identified for each task.

## 7. Experiments

The proposed framework was systematically tested on two datasets: the Environmental Constraint Exploitation (ECE) dataset (Puhlmann et al., 2016) and the EgoHands dataset (Bambach et al., 2015). The ECE dataset represents the ideal test bed for evaluating the network capabilities in correctly identifying the hand in the video frames, and classifying the dynamic action sub-components (or atomic behavior) that compose the entire manipulation act. The EgoHands dataset, instead, is used to verify the effectiveness of our approach in recognizing a whole meaningful task-oriented video-recorded action, and it is employed to comparatively assess the performance of our method with state of the art techniques.

### 7.1. ECE Dataset: Motivation

In robotic grasping and manipulation, many datasets have recently been created by a number of groups for different research purposes and have been shared with the robotics community (Huang et al., 2016). However choosing the right dataset is not an easy task, since it depends on which is the purpose we would like to achieve.

Since human grasping capabilities are definitely superior to robotic ones, one challenge is to investigate the principles of human grasping and work toward transferring these principles to robotic systems. In the paradigm of soft robotic manipulation, where soft yet adaptable artificial hands deform to shape around external object, the leading hypothesis is that successful grasping performance crucially depends on the purposeful *exploitation* of contact with the environment. More specifically, the environment provides *environmental constraints* (ECs) (Eppner et al., 2015; Averta et al., 2017, 2018; Della Santina et al., 2017, which allow to relax the requirements for accurate control, simplify perception, and facilitate grasp planning. The dynamic aspects resulting from the interaction between hand, object, and environment are essential for human grasps and may represent an unmatched source of inspiration for devising successful and robust approaches to soft robot grasping. To favor such a cross-fertilization between neuroscientific observations and robotics research, our research aims at analyzing videos on human hands during ECE-based object grasping, to identify dynamic strategies for a successful “manipulation with the environment” task. To this end, we use the fully human labeled raw videos provided in Puhlmann et al. (2016), where each atomic behavior or action primitive represented in the frames was human-labeled based on a dictionary described in the following subsections, representing an ideal, test bench for what concerns the classification of dynamic aspects in hand centered strategies.

#### 7.1.1. Video Description

Videos capture single-object table-top human grasps. In each video, the participant grasps one out of twenty five different objects placed in front of him and lifts it. The objects were chosen so as to favor different grasping actions involving a purposeful interaction with the environment, as reported in Heinemann et al. (2015). At the trial onset, each participant placed their dominant hand at a given and predefined starting position on the table. After an audio signal, the participant initiated the grasp. Only the dominant (right) hand was involved in the task accomplishment. Video recordings started with the audio signal and ended once the object was lifted. Our dataset is composed by experimental videos acquired from ten participants, 150 videos for each participant, for a total of 1,500 videos.

#### 7.1.2. Video Segmentation and Labeling

Assuming that humans are quite proficient in recognizing actions of other people's motion, videos were manually segmented and labeled using the action primitives defined below. A graphical user interface was used to facilitate annotation in Puhlmann et al. (2016). Each grasping video is represented by a combination of six different action primitives: *rest, approach, close, slide, flip, edge*. In the following, we briefly describe the conditions for the action primitives.

- *Rest/Stay* the participant's hand is in rest position;
- *Approach* the participant moves his hand to reach the object, the action lasts until the hand makes contact with the object;
- *Close* the fingers start flexing around the object, the action lasts until the fingers fully wrap the object or when the object loses contact with the table;
- *Flip* the fingers has already wrapped the object which rotates around one of its edges in contact with the table, the action lasts until the object loses contact with the table;
- *Slide* the participant begins to reorient or reposition the object on the table surface, the action lasts until the object motion slows down and another action begins;
- *Edge* such action usually follows the *slide* action, more specifically, the object is already at the edge of the table and participant pulls it over the edge to reveal its bottom side, the action lasts until the object loses contact with the table.

#### 7.1.3. Video Frame Pre-processing

While the CNN was trained on RGB images (hands), our videos instead are in black and white. Furthermore the participants wore a glove during the experiments. We thus convert each frame into RGB by applying a simple image segmentation filter using the *imbinarize* function in MatLab (based on *Otsu's method*; Otsu, 1979). In this way we can select the pixels connected to the glove, which roughly represent the hand, and colorize them with the same color, chosen as the mean value of EgoHands ground truth (Bambach et al., 2015).

#### 7.1.4. Training Details

To execute the *Hand features extraction* pipeline, we used pre trained models (Bambach et al., 2015) for both window proposals generation and classification.

The *Sequence learning* network was instead trained from scratch. We used *keras library* (Chollet et al., 2015) for LSTM network implementation, training, and testing. The library is written in Python and uses Theano (Bergstra et al., 2010) as backend. Keras supports GPU, which makes the training time up to 1000 times faster than training with a CPU. All the procedures were implemented on a NVIDIA Tesla M40 GPU with 12GB of onboard memory. The datasets we considered are large enough to minimize the risk of overfitting, which was further reduced through the usage of the dropout technique (Srivastava et al., 2014).

#### 7.1.5. Evaluation on ECE Dataset

To verify the generalization and robustness of action primitive classification, we examined two forms of cross-validation: *hold out* and *leave one out*. The goal of cross-validation is to estimate the expected level of model predictive accuracy in way that is independent from the data used to train the model.

First, we have used a simple hold out cross validation to identify hyper-parameters. We randomly split our dataset in: 70% video clips for training, 20% for validation and 10% are reserved for testing. We trained 50 different network configurations which toke into consideration the most relevant model hyper-parameters, such as network architecture (i.e., number of hidden layers and number of LSTM cells per layer) and learning hyper-parameters (i.e., learning rate, dropout, number of epochs and the batch size). The training time for each network configuration ranged from 4 to 8 h. Looking at the results of each simulation, we select the configuration, which provided both the highest *min-score* and *f1-score* accuracy on the validation dataset − which are 73 and 91% respectively. In such configuration, we use three hidden layers, with respectively 256, 256, and 128 dimensions for the size of the LSTM memory. We train the network for 30 epochs using RMSprop optimizer (to avoid the gradient vanishing/exploding issues) (Tieleman and Hinton, 2012) with a fixed learning rate of 0.001 and no momentum. The batch size is set to 20, and all weights are randomly initialized. The temporal reference length *n* of videos is actually a hyper parameter. We consider only video frames that contains hands detected by the CNN, hence, each video is characterized by a total number *n* of feature vectors ranging from 30 up to 120 (videos last between 2 and 8 s with 15 frames per second). To compute the hyper parameters of the network we first fixed *n* = 60 (the mean value). Generally the number of the feature vectors representing a generic video is *p* ∈ [30, 120]; if *p* > 60 (the video is too long) we select uniformly 60 feature vectors, otherwise *p* < 60 (the video is too short) we pad 60−*p* vectors at the beginning of the sequence. Once we selected all the hyper parameters of the network, we empirically chose the temporal reference length of videos (number of hand feature vectors representing the entire video). We tested different values of *n* ranging between 30 and 120 (i.e., 30, 40, 50, 60, 70, 100, 120). Based on the results of each simulation we select *n* = 50. Table 2 shows the *min-score* accuracy (the lowest accuracy with respect to the six classes) and *f1-score* (Yang and Liu, 1999) accuracy on the validation dataset in function of hyper-parameter settings. With such architecture and such learning and training parameters, the network is able to classify the dynamic hand strategies in the test dataset with an accuracy ranging from 75% up to 96%, depending on the action class. Normalizing scores with respect to the total number of classes, we obtain an accuracy of 85%.

**Table 2:** *Min-score* and *f1-score* accuracy on the validation dataset depending on hyper-parameter settings.

***LSTM_**1**_***	***LSTM_**2**_***	***LSTM_**3**_***	***Batch***	***Epochs***	***Dropout***	***n***	***Min-score***	***F1-score***
64	64	–	10	20	0.3	60	63.3	85.1
128	128	–	10	20	0.3	60	65.2	86.4
256	256	–	10	20	0.3	60	64.7	88.3
512	512	–	10	20	0.3	60	64.1	86.6
256	256	64	10	20	0.3	60	66.2	88.4
256	256	128	10	20	0.3	60	68.2	90.3
256	256	256	10	20	0.3	60	68.1	88.6
256	256	128	20	20	0.3	60	70.8	89.7
256	256	128	20	30	0.3	60	71.7	90.6
256	256	128	20	30	0.5	60	71.8	91.1
256	256	128	20	30	0.5	30	68.1	87.4
256	256	128	20	30	0.5	40	70.2	90.6
256	256	128	20	30	0.5	70	69.6	89.8
256	256	128	20	30	0.5	100	67.3	85.4
256	256	128	20	30	0.5	120	65.4	85.1
**256**	**256**	**128**	**20**	**30**	**0.5**	**50**	**73.4**	**91.3**

*LSTM_i_ is the number of LSTM cell units relative to the hidden layer i, batch is the number of training examples in one forward/backward pass, epochs describe the number of times the algorithm sees the entire training dataset, dropout is the parameter of dropout method (Srivastava et al., 2014), and n is the input sequence length (number of hand feature vectors representing the entire video). The last row in bold refers to the configuration we select, which provides both the highest min-score and f1-score accuracy*.

Although on-line classification is not the goal of this work, since our objective is to deeply characterize human grasping primitives for a possible translation on the robotic side, the *sequence learning* network we propose could be in principle be used also for real time predictions, since it requires less than 2 ms to process each hand feature vector. Therefore, such real time hand action predictions could be employed as additional information during planning of a soft robot manipulator. We will further evaluate this aspect in future works.

**Figure 5:**
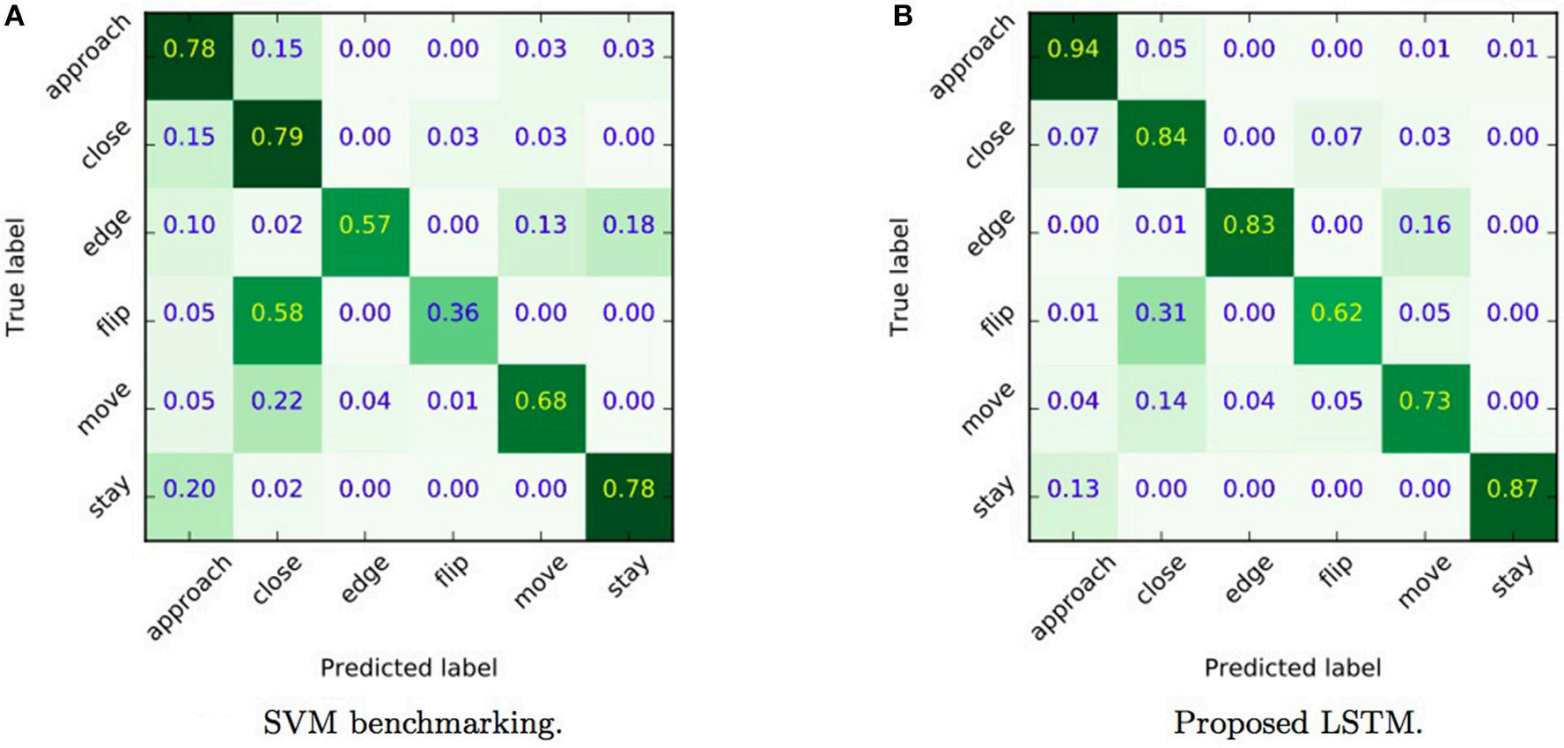
Normalized confusion matrices relative to the accuracy of a benchmark SVM method **(A)** and the proposed LSTM **(B)**. Showing per class precision on the diagonal, true classes on the y-axis and predicted classes on the x-axis. As we can observe the accuracy with the SVM is significantly lower than the one observed with our approach allowing us to confirm that the inclusion of temporal information for action primitive classification is crucial.

We also carried out cross-validation analyses of network performance, which require a longer computation time compared to hold out procedure, but ensure more robust validation outcomes. The training and evaluation time lasted 20 days on a NVIDIA Tesla M40. Figure 5B shows the per class normalized confusion of the classifier of all 6 classes that were detected. We note that there is a class (*approach*) with a precision of over 94%, three classes with a precision over 83% (*edge, close, rest*), one class (*slide*) with a precision of 73% and one (*flip*) that reaches a reasonable 62%. This is a very strong result given the fact that the classifier is trained on videos with poor quality − videos are in black and white format, and subjects wore gloves. Still, the question remains why the approach does not recognize all action primitives equally well? Some possible reasons include: (i) labels in the dataset are not equally distributed across the classes, leading to a recognition bias, and (ii) the highly dynamical and complex manipulation strategies introduce differences with respect to the hand shape taxonomies used for creating the action primitive labels of the dataset (even though an experienced person performed it).

With the aim of observing how the accuracy changes if no temporal information is considered in the classification, we tested an SVM model Cortes and Vapnik (1995), implemented through the *scikit-learn* library in python Pedregosa et al. (2011). SVM is a state of the art method then it comes to classifying static vectors with no particular bias or structure. We trained an SVM classifier equipped with an RBF kernel to classify each hand feature vector considering the aforementioned six action primitives. A leave one out cross-validation is used to select SVM hyper-parameters: the results corresponding to the selected model configuration are depicted in Figure 5A. Normalizing scores with respect to the total number of classes, we can observe that the accuracy with the SVM is significantly lower (66%) than the one observed with our approach reported in Figure 5B (80%), revealing that the inclusion of temporal information for action primitive classification is crucial.

**Figure 6:**
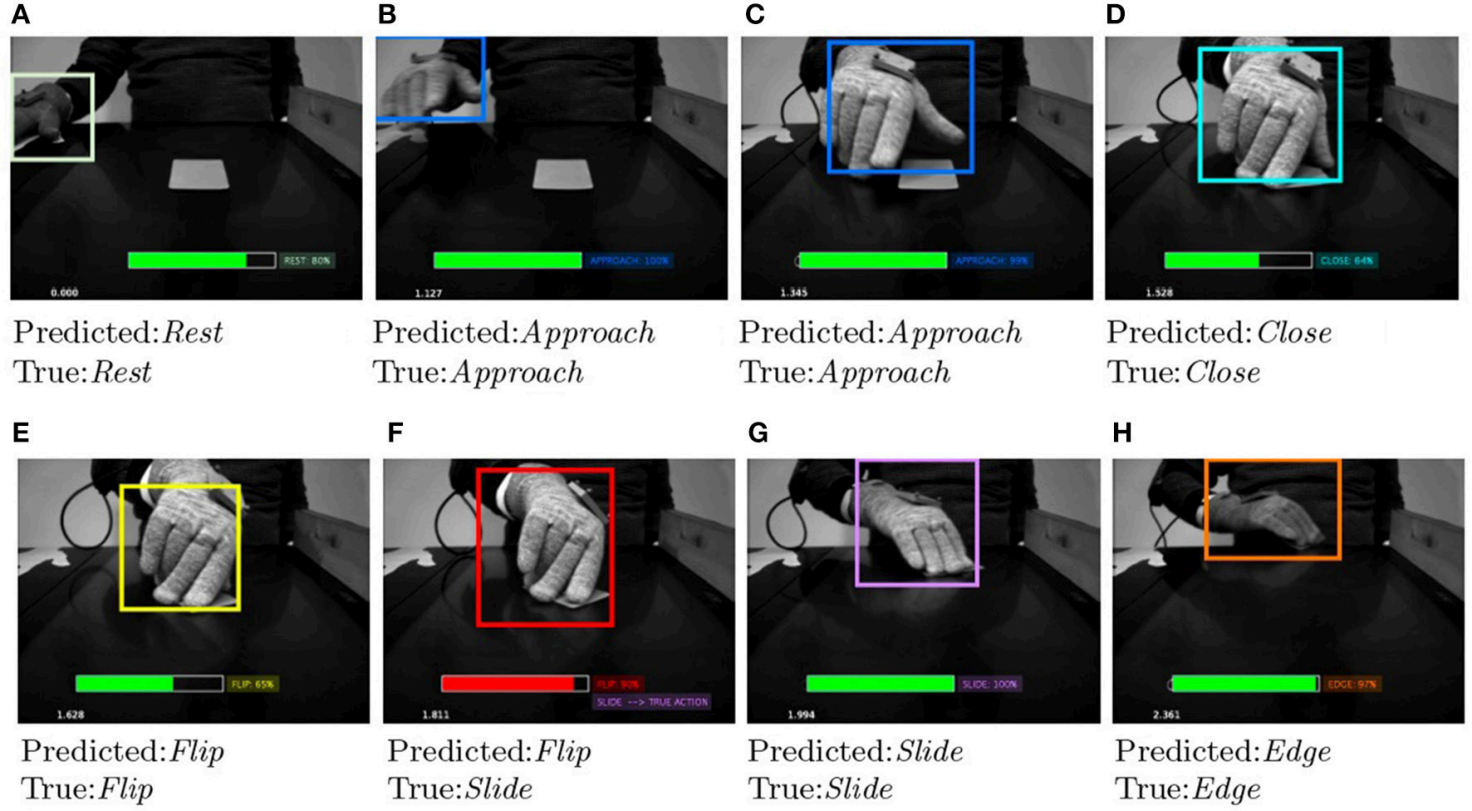
Evolution of some salient action primitives the network identifies in the reaching and grasping a credit card. In each video frame we overlaid (i) the bounding box which likely enclose the hand, color changes depending on the action primitive the hand currently performs, (ii) the predicted action label, and in case of failure, the true label, and (iii) the level of confidence represented with a filled rectangle, color is green if the predicted action is true **(A–E,G,H)**, red otherwise **(F)**.

**Figure 7:**
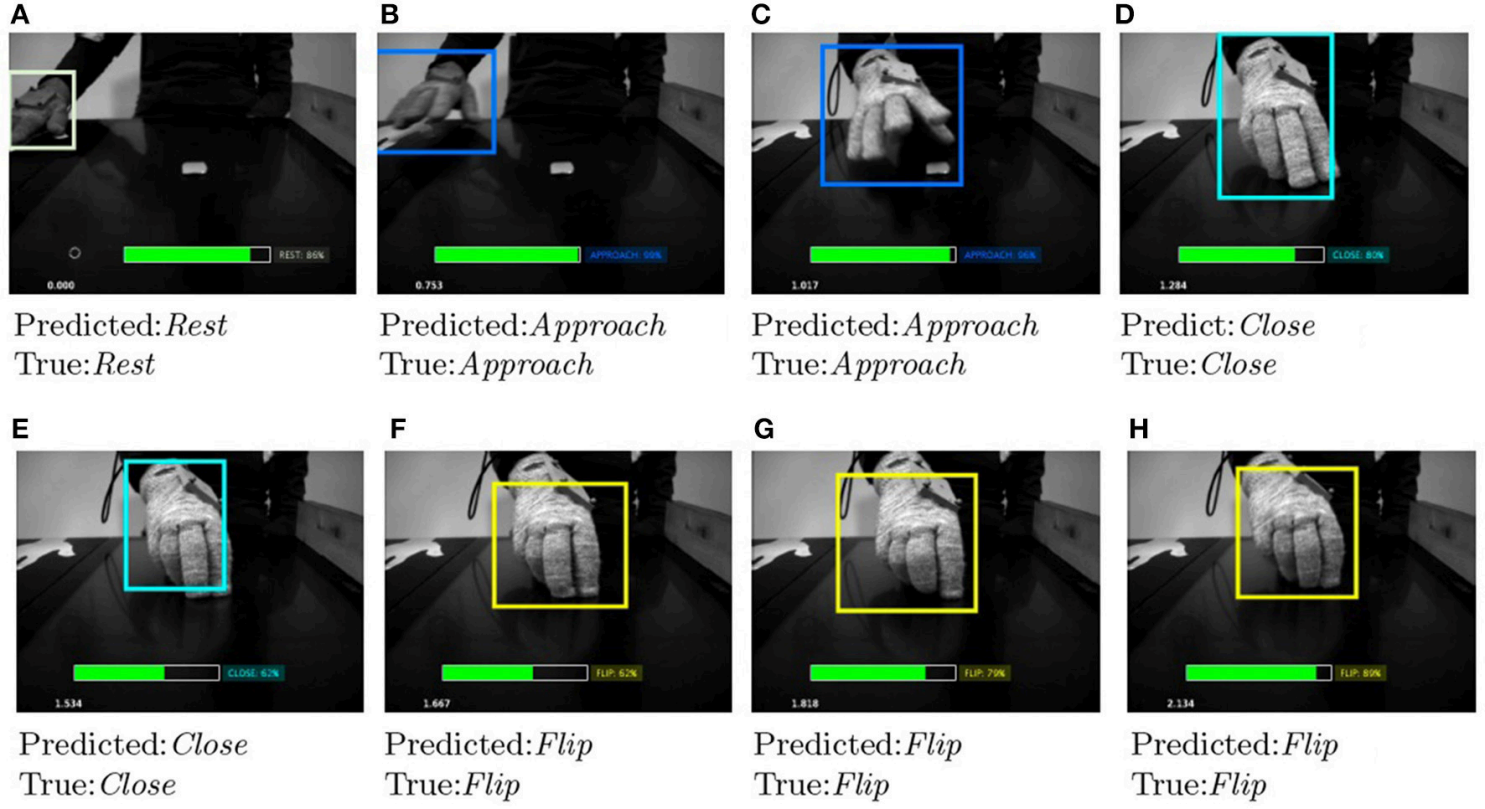
Evolution of some salient action primitives the network identifies in the reaching and grasping a French chalk. In each video frame **(A–H)**, we overlaid (i) the bounding box which likely enclose the hand, color changes depending on the action primitive the hand currently performs, (ii) the predicted action label, and (iii) the level of confidence represented with a filled rectangle.

Figures 6, 7 show some examples of action primitives identified by our DeepDynamicHand model on ECE dataset. In each video frame we overlaid (i) the bounding box which likely enclose the hand, color changes depending on the action primitive the hand currently performs, (ii) the predicted action label, and in case of failure, the true label, and (iii) the level of confidence represented with a filled rectangle, color is green if the predicted action is true, red otherwise. We can observe that wrong classification usually happens when a transaction between primitives occurs. Frame Figure 6F refers to such example, showing that confusions occurs in situations where hand shapes are hardly distinguishable also by a human.

**Figure 8:**
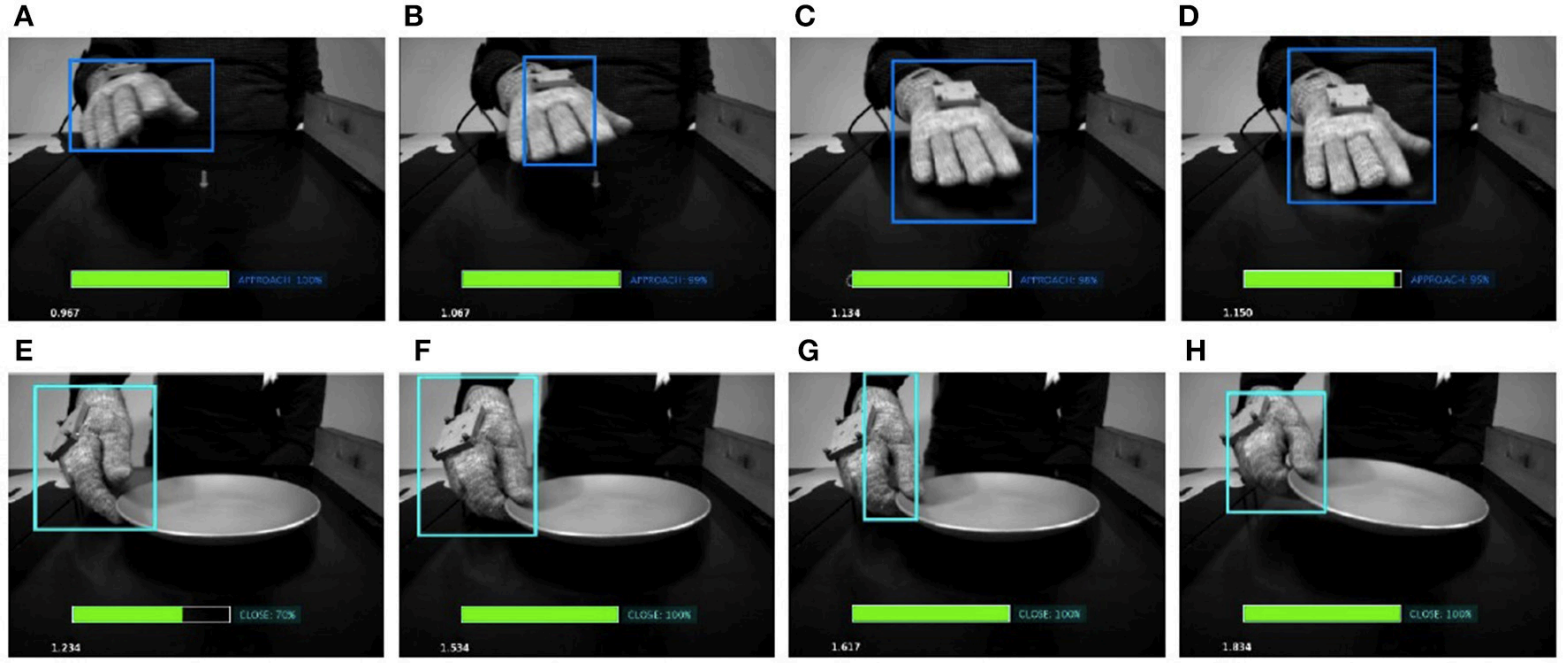
Sequence of video frames when partially detected hand occur **(B,G)**. In order to evaluate the correctness of the sequence learning network, we include some prior and posterior frames **(A,C,D)**, and **(E,F,H)**, respectively. We can observe that in such situations the LSTM network is able to correctly predict the right action primitive.

**Figure 9:**
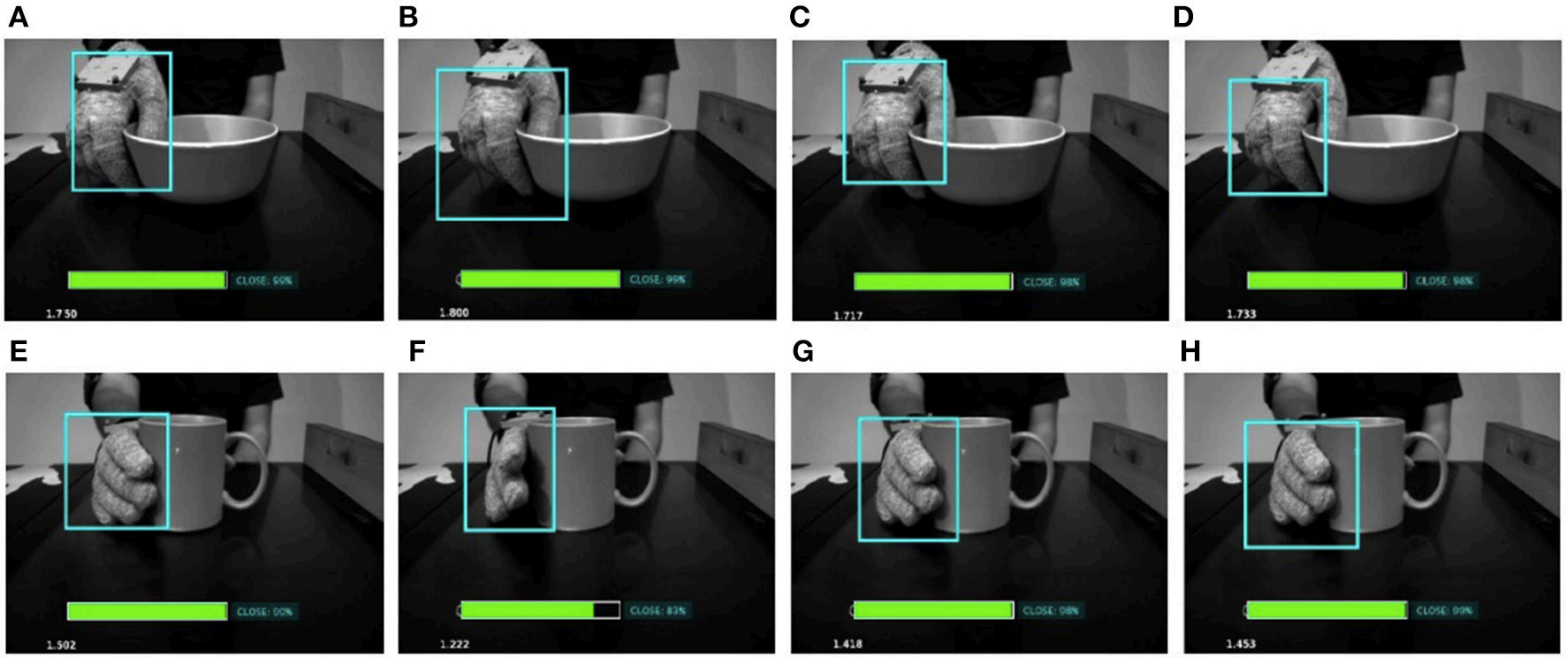
Some salient video frames with partially occluded hands. In **(A–D)**, the hand is occluded by a bowl, while in **(E–H)** by a coffee mug. We can observe that in such situations the LSTM network is able to correctly predict the right action primitive.

It is worth to note that despite the pre trained network we used to detect the hand works very well, there may be situations where it captures hands only partially. We trained the LSTM network using the partially detected hand features, making the model more robust and able to correctly predict the right primitive despite partial hand recognitions. In Figures 8, 9 show some salient video frames with partially detected hands‘ and partially occluded hands, respectively. We can observe that in these situations the LSTM network is able to correctly predict the right action primitive.

### 7.2. EgoHands Dataset

As already discussed in section 2, EgoHands (Bambach et al., 2015) uses a second CNN to classify whole labeled video frames choosing one out four. Hence, only one label is attached to each video. We tested the same DeepDynamicHand in this scenario, to compare its performance with respect to the state of the art benchmarking provided by the second CNN implemented in Bambach et al. (2015). The goal is to show the effect of including temporal dynamic information as well as to test the robustness of the network with respect to the different perspectives taken from the subjects' own viewpoint.

#### 7.2.1. Data Collection

**Figure 10:**
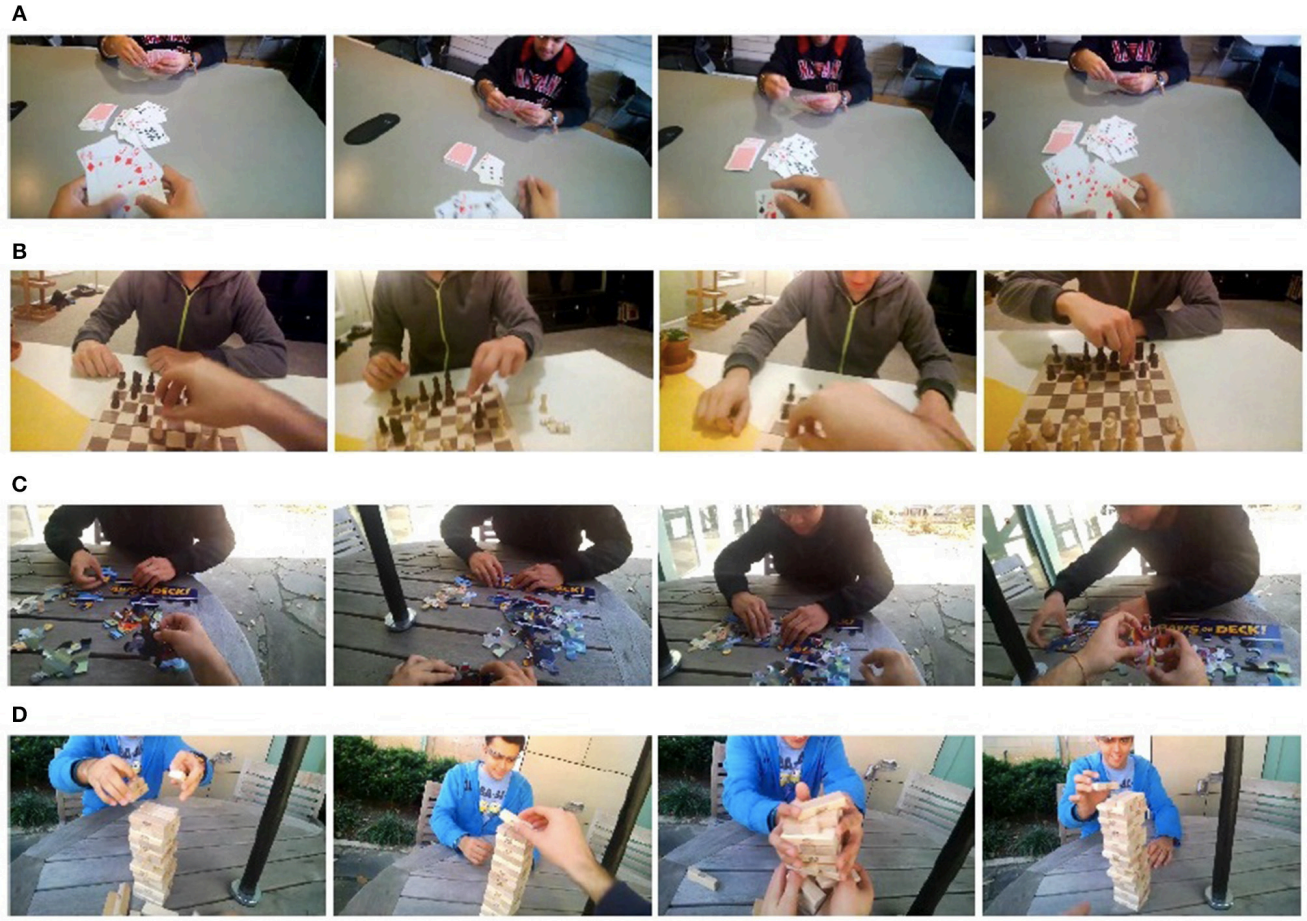
Some video frames relative to EgoHands dataset (Bambach et al., 2015) of people performing four types of activities: playing cards **(A)**, playing chess **(B)**, solving a puzzle **(C)**, and playing Jenga **(D)**, respectively. Unlike the ECE dataset, in such videos subjects do not wear glove and acquisitions are captured from a wearable first-person camera, which provides egocentric images (perspectives taken from the subjects' own viewpoint).

The dataset contains 48 first-person videos of people performing four types of activities (playing cards, playing chess, solving a puzzle, and playing Jenga) Figure 10. Such dataset consists of around 130,000 frames of video, 4,800 of which have pixel-level ground truth consisting of 15,053 hands.

#### 7.2.2. Evaluation on EgoHands

The task is to classify whole video as one of four different activities, still exploiting hand pose information, without using any information about the appearance or identity of handled objects or the rest of the scene. In Bambach et al. (2015) the CNN is trained on their ground truth dataset (where each frame is represented in terms of player's segmented hands) to make a frame-by-frame classification. Then a simple voting-based approach is used to incorporate temporal information: each individual frame is classified in the context of a fixed-size temporal window centered on the frame. In other words, scores across the window are summed, and the frame is labeled with the highest scoring class.

**Figure 11:**
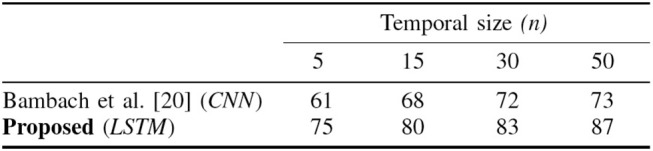
Activity recognition accuracies relative to Bambach et al. (2015) approach and the proposed LSTM method. The proposed LSTM network increases significantly the accuracy exploiting in a more efficient way the temporal dependencies.

To make a comparison, we evaluate our framework on the same ground truth EgoHands dataset. We extract hand features for each frame, then combine them in order to create input sequences. To take into account temporal information we exploit only LSTM capabilities (whose architecture is the same as the one used in ECE dataset). To make our results comparable with the ones provided in Bambach et al. (2015), we change the input sequence length *n* in function of the number of frames we currently considered (as reported in Figure 11). To evaluate our model we use leave one out cross-validation. In Figure 11 we can observe that temporal information − i.e., the number of video frames considered − is beneficial in increasing activity recognition accuracy. In particular the proposed LSTM network increases significantly the accuracy (87%) since it exploits in a more efficient way the temporal dependencies.

## 8. Impact in Robotics

The description of human grasping as sequences of action primitives can provide a useful tool to extract transition-based grasping plans for soft robotic manipulators. To devise robotic manipulation strategies that involve a thorough interaction with the environment, getting insights from the human example could be the successful strategy to design bio-aware robots that can fully take advantage from ECE, like humans do. In this way, we could transfer both the action primitives and the ability to decide on the most appropriate transitions between them Krishnan et al. (2017). In Puhlmann et al. (2016) authors argued that the analysis of human grasping behavior in terms of transition probabilities between primitives allows us to identify the conditions that determine the decision for one path of action over another, and to specify the conditions these transitions depend on. Based on their data analysis, they observed that human subjects can reliably use different action sequences for different object shapes. By suitably replicating these observations on the artificial side, we could enable a robot to correctly evaluate, the transition conditions (so as to comply with the sequence of action primitives relative to each learned sequence) by means of appropriate tools and sensors. Let us say we start with *approach*. To make the robot capable of choosing between *close, flip* or *move*, we can exploit another CNN that we can train in order to recognize the orientation and the shape of the object. With the addition of a position sensor, the robot could be also able to perform the *edge* grasp or not. Generally robot manipulators (i.e., KUKA LWR arm) we can use to implement the human-like primitives have more degrees of freedom (DOFs) than required. Hence, an important aspect is the planning of such redundant robot arms since the redundancy of course improves their performance (e.g., obstacle avoidance, singularity avoidance, and so on) but on the other hand it allows for infinite solutions corresponding to a specific position of the end-effector (i.e., robust yet deformable robotic hands). The inverse kinematic problem is one of the challenging issues for robot redundant manipulators and the traditional ways to solve it (e.g., analytical solutions or pseudoinverse-based methods) could come with joint-angular-drift problems Klein and Kee (1989). We can exploit one of the recent varying-parameter neural networks proposed in Zhang et al. (2018a,b,c) to cope with the joint-angular-drift problems.

## 9. Conclusions

The framework we implemented is capable of automatically characterizing the sequences of action primitives that underpin meaningful task-oriented hand actions. Our approach, which combines CNN and RNN, is the first attempt to include also dynamic information for classifying different time related action primitives, human use for grasping and manipulation tasks. This is done by exploiting only the hand pose features extracted from video frames, without considering further information about the environment constraints and the shape of the objects, despite the fact that action primitives heavily depend on these. We tested our approach on two datasets: one that includes videos where participants interact with the environment to grasp the objects, with the goal of identifying dynamic atomic behavior composing the whole task execution; the other one, involving videos about daily living activities, where the objective is to identify a complete meaningful action from the whole video. Results show that, considering temporal information in the classification procedure, the performance of our technique − normalized with respect to the total number of classes − is 80% on ECE dataset and 87% on EgoHands dataset, outperforming other state of the art methods that do not taken into account action dynamics. We discuss how these results can be used in robotics, to transfer human capabilities onto a successful design of robotic systems. Future works will be devoted to further push our approach for robot planning and development. We will also use the here proposed architecture to automatically label videos taken from the internet. The labeled results will be exploited to train a deep neural network for autonomous generation of grasping strategies with robot manipulators.

## Author Contributions

All the authors contributed in defining the proposed architecture. VA and CD performed experiments. VA, CD, DB, and MB performed the data analysis. All the authors contributed to writing the manuscript.

### Conflict of Interest Statement

The authors declare that the research was conducted in the absence of any commercial or financial relationships that could be construed as a potential conflict of interest.
